# Four Spices Differentially Modulate Roasted Beef Patty Quality and Fluorescent ALEs Formation via Carbonyl Precursors

**DOI:** 10.3390/foods15173023

**Published:** 2026-08-27

**Authors:** Yaya Wang, Yuanfei Li, Tianchang Zhang, Bingxin Yang, Yiming Zhang, Yuekun Wu

**Affiliations:** 1 College of Food and Health, Zhejiang A&F University, Hangzhou 311300, China; 18829349424@163.com (Y.W.); liyuanfei2024@163.com (Y.L.); 2 School of Medicine, Nankai University, Tianjin 300071, China; cb15939525699@163.com; 3 College of Optical, Mechanical and Electrical Engineering, Zhejiang A&F University, Hangzhou 311300, China; 2025612041010@stu.zafu.edu.cn

**Keywords:** spices, advanced lipid oxidation end-products, malondialdehyde, antioxidant activity, lipid oxidation

## Abstract

The accumulation of dietary advanced lipid oxidation end-products (ALEs) in thermally processed meat products is closely associated with quality deterioration and potential health risks. Spices are widely recognized for both flavoring and antioxidant properties and may therefore help reduce ALEs formation, yet their comparative and dose-dependent effects remain insufficiently defined. In this study, roasted beef patties were used as a model food to systematically evaluate the effects of dried tangerine peel, cinnamon, clove, and rosemary, each individually added at 0.1%, 0.5%, and 1.0% (*w*/*w*), on product quality, ALEs formation and the accumulation of ALEs precursors. Pearson correlation analysis and principal component analysis were further applied to explore the relationships among spice antioxidant properties, carbonyl precursor accumulation, and ALE-associated fluorescence. The results showed that the four spices had no significant effect on cooking loss but influenced color and, to a lesser extent, texture attributes. Their inhibitory effects on ALEs formation differed substantially. Clove and rosemary showed stronger and more stable inhibition, particularly at 0.5% and 1.0% addition levels. Precursor analysis showed that clove and rosemary markedly reduced malondialdehyde (MDA) accumulation, whereas glyoxal and methylglyoxal exhibited more variable responses among treatments. Changes in MDA were more consistent with those in ALE-associated fluorescence. Clove exhibited the highest total phenolic and flavonoid contents and overall antioxidant potential. Integrated analysis further indicated that stronger antioxidant properties were associated with lower MDA levels and ALE-associated fluorescence. However, the relatively strong in vitro antioxidant activity of cinnamon did not translate into stronger inhibition in the beef patty matrix, suggesting that antioxidant capacity alone could not fully explain the spice-dependent effects. Overall, clove and rosemary at 0.5–1.0% (*w*/*w*) were the most promising treatments for mitigating fluorescent ALE-associated product formation in roasted beef patties.

## 1. Introduction

Meat is an important source of high-quality protein, lipids, vitamins and minerals in the human diet. With the development of food culture and processing technologies, meat products are now prepared using a wide range of methods, including frying, deep-frying and roasting in Western cuisines, as well as steaming, boiling, stewing and braising in Chinese culinary traditions. Among these methods, roasting can rapidly generate desirable color, flavor and texture attributes in meat products [1], and therefore plays an important role in global food culture.

The sensory quality of roasted meat is driven by multiple chemical reactions under high-temperature conditions. On the one hand, the Maillard reaction between proteins and reducing sugars produces melanoidins, heterocyclic compounds and other flavor-active substances, contributing to the characteristic golden to brown color. On the other hand, lipid oxidation and the resulting volatile compounds further shape the typical aroma of roasted meat. In addition, marination and thermal processing induce moderate denaturation of myofibrillar proteins and redistribution of water, thereby improving the final texture of meat products [2,3]. Roasting is therefore not only a traditional cooking method, but also an important processing technology in the modern meat industry because it can balance flavor development and nutritional value.

However, high-temperature roasting also promotes complex interactions among lipids, proteins and carbohydrates, leading to the formation of multiple processing-induced hazards. Lipid oxidation is one of the key reaction pathways during roasted meat processing [4]. Meat products are typical high-fat and high-protein food systems. During roasting, unsaturated fatty acids first form primary oxidation products, such as hydroperoxides, which then undergo oxidative cleavage and rearrangement to generate highly reactive secondary carbonyl compounds, including malondialdehyde (MDA), 4-hydroxynonenal, glyoxal (GO) and methylglyoxal (MGO). MDA and 4-hydroxynonenal are predominantly generated through lipid peroxidation, whereas GO and MGO may arise from both lipid oxidation and carbohydrate-related pathways, including sugar degradation and Maillard reactions. Lipid oxidation may nevertheless represent an important source of GO and MGO in lipid-rich meat matrices exposed to high temperatures.

During thermal processing, reactive carbonyl compounds covalently modify nucleophilic amino acid residues through compound-specific reaction pathways. MDA primarily forms Schiff-base adducts and protein cross-links involving lysine residues, whereas GO and MGO preferentially react with lysine and arginine. In contrast, α,β-unsaturated aldehydes, such as 4-hydroxynonenal, may modify cysteine, histidine and lysine residues through Michael addition. These reactions generate a series of structurally heterogeneous carbonyl–protein adducts and cross-linked products known as advanced lipoxidation end products (ALEs) [5,6,7]. Previous studies have shown that ALEs are readily formed in meat products with high lipid and protein contents, especially under high processing temperatures and prolonged heating. ALEs formation not only contributes to the deterioration of flavor and color, but also reduces nutritional value and may raise health concerns. Carbonyl modification can markedly alter protein structure by inducing conformational unfolding, aggregation and cross-linking, while also blocking digestive enzyme cleavage sites. These changes reduce protein digestibility and the bioavailability of essential amino acids, particularly lysine [8,9,10]. Carbonyl-derived protein adducts and cross-linked structures may further restrict protease accessibility and thereby decrease protein digestibility [11,12].

Dietary ALEs may also exert broader biological effects after ingestion through digestion, absorption, tissue distribution and metabolic responses. Epidemiological evidence has associated high intake of thermally processed foods with cardiovascular disease, metabolic syndrome and other health disorders. One possible contributor is the formation of reactive carbonyl compounds and their modified proteins during food thermal processing. In recent years, growing evidence has linked dietary ALEs to oxidative stress, chronic inflammation, disrupted energy metabolism, liver injury and impaired cognitive function. Our previous studies also showed that short-term intake of ALEs induced liver dysfunction and lipid deposition in mice, whereas prolonged intake increased blood glucose, aggravated insulin resistance and worsened liver injury. In vitro experiments further showed that ALEs significantly reduced ATP levels in hepatocytes, leading to impaired energy homeostasis. These findings suggest that ALEs are not only markers of food quality deterioration, but also potential lipid oxidation-derived hazards in thermally processed meat products [13].

Reducing lipid oxidation and protein carbonyl modification while maintaining the flavor and quality of roasted meat is therefore an important goal in the study of thermally processed meat products. Current mitigation strategies include optimizing processing parameters, applying milder cooking methods and adding natural bioactive ingredients such as vitamins, plant extracts, spices and herbs [14,15,16,17]. Spices have attracted particular attention because they provide both flavor and antioxidant activity, making them potential natural modulators of processing-induced hazard formation [18]. Spices and their extracts can influence the formation of heterocyclic amines, advanced glycation end-products and lipid oxidation products [19,20]. Possible mechanisms include free radical scavenging, reactive carbonyl trapping and protein–polyphenol interactions [21,22]. However, the reported effects vary widely because of differences in spice form, food matrix and processing conditions. In some systems, certain spices may even promote the formation of specific thermal reaction products [23]. Although relevant evidence remains limited, previous studies have investigated the potential of herbs and spices to control lipid oxidation and related processing hazards in beef systems. Van Hecke et al. [24] demonstrated that the effects of ten herbs and spices on the formation of MDA, 4-hydroxy-2-nonenal and hexanal during heating and in vitro gastrointestinal digestion of a beef product varied with spice type, addition level and application stage. Yu et al. [25] further reported that rosemary, turmeric and bay leaf inhibited the formation of MDA, GO, MGO and several other processing-induced hazards in roasted beef patties while also affecting product quality. However, these studies mainly focused on lipid oxidation products or multiple small-molecule hazards and did not evaluate fluorescent ALE-associated product formation. Therefore, the comparative and dose-dependent effects of spices on fluorescent ALE-associated product formation, as well as its relationships with carbonyl precursor accumulation, spice antioxidant properties and product quality, remain insufficiently understood.

In this study, roasted beef patties were used as a model system to investigate the effects of four representative spices, namely dried tangerine peel, cinnamon, clove and rosemary, on ALEs formation and product quality during roasting. Each spice was added at three levels: 0.1%, 0.5% and 1.0%. These spices were selected because they represent chemically distinct plant matrices containing different profiles of phenolic and other antioxidant constituents; however, their comparative and dose-dependent effects on ALEs formation in roasted meat remain unclear. Because ALEs are structurally diverse and chemically complex, there is currently no widely accepted quantitative method that can cover all ALEs in food systems. Existing targeted methods mainly quantify several representative MDA-derived protein adducts, but these methods reflect only specific ALEs species and cannot represent total ALEs accumulation. Given that some lipid oxidation-derived protein modification products exhibit characteristic fluorescence, this study referred to the evaluation strategy used for fluorescent advanced glycation end-products and adopted the excitation and emission wavelength ranges identified in our previous experiments. Bound fluorescence intensity was therefore used to characterize the relative accumulation of fluorescent ALEs in roasted beef patties.

On this basis, we compared the effects of different spices and addition levels on ALE-associated fluorescence and evaluated their effects on cooking loss, color and texture. We further measured representative reactive carbonyl compounds associated with ALEs formation, including MDA, GO and MGO, and analyzed the relationships among spice antioxidant properties, carbonyl accumulation and ALE-associated fluorescence. This study aimed to clarify the spice- and dose-dependent regulation of fluorescent ALEs formation and to determine whether the mitigation of carbonyl-related hazards could be achieved without adversely affecting the quality of roasted beef patties.

## 2. Materials and Methods

### 2.1. Materials and Reagents

Commercial food-grade dried tangerine peel, dried rosemary leaves, cinnamon bark, and clove buds were purchased from local retailers in Tianjin, China. Beef tenderloin was purchased on three separate occasions from a China Resources Vanguard supermarket in Jinnan District, Tianjin, China. The raw material was commercially available bovine tenderloin, corresponding primarily to the psoas major muscle. Because the beef was purchased through a commercial retail supply chain, animal-specific information such as breed, sex, age, and slaughter history was not available. After purchase, the beef was transported to the laboratory under refrigerated conditions, trimmed of visible fat and connective tissue, and stored at 4 °C until processing. Before treatment allocation, the pH of each raw-beef batch was measured at three locations using a calibrated portable pH meter (DELTA 320, Mettler-Toledo Co., Ltd., Shanghai, China). Representative samples from each batch were homogenized and analyzed in triplicate for crude protein and crude fat. Crude protein was determined by the Kjeldahl method using a nitrogen-to-protein conversion factor of 6.25, and crude fat was determined by Soxhlet extraction. The raw beef had a mean pH of 5.63 ± 0.24 and contained 21.72 ± 0.58% crude protein and 3.68 ± 0.96% crude fat on a wet-weight basis. Results are expressed as the mean ± standard deviation of three independent beef batches.

Sunflower oil was supplied by JiaGe Investment (China) Co., Ltd. (Hong Kong, China). Hydrochloric acid and absolute ethanol were purchased from Tianjin Chemical Reagent No. 1 Factory (Tianjin, China) and Tianjin Chemical Reagent No. 6 Factory (Tianjin, China), respectively. Acetonitrile, n-hexane, and methanol, as well as sodium hydroxide, were purchased from Sigma-Aldrich (St. Louis, MO, USA). Thiobarbituric acid was purchased from Shanghai Macklin Biochemical Co., Ltd. (Shanghai, China), whereas potassium chloride (analytical grade) was purchased from Sinopharm Chemical Reagent Co., Ltd. (Shanghai, China). Ultrapure water was produced using a Milli-Q water purification system (EMD Millipore Corporation, Burlington, MA, USA). C18 solid-phase extraction (SPE) sorbent (particle size, 40–63 μm) was purchased from Shanghai ANPEL Scientific Instrument Co., Ltd. (Shanghai, China). Oasis HLB cartridges (3 mL, 60 mg) were purchased from Waters (Milford, MA, USA). Organic-solvent-compatible membrane filters (0.22-μm pore size) were purchased from Tianjin Jinteng Experimental Equipment Co., Ltd. (Tianjin, China). o-Phenylenediamine, DPPH, ABTS, Folin–Ciocalteu reagent, gallic acid, and rutin were purchased from Sigma-Aldrich (St. Louis, MO, USA).

### 2.2. Preparation of Beef Patties

Beef tenderloin was purchased on three separate occasions from a China Resources Vanguard supermarket in Jinnan District, Tianjin, China. Each purchase constituted one independent raw-material batch and was regarded as one independent biological replicate. Visible fat and connective tissue were removed, and the meat was cut into small pieces. The meat was then ground once through a 5 mm plate using a meat grinder and thoroughly mixed. The ground meat was manually mixed for approximately 1 min to obtain a uniform base meat matrix before allocation to the different treatments. For each batch, patty formulation, marination, roasting, post-roasting sample processing, storage, and subsequent analyses were conducted independently. Within each independent replicate, the minced beef was assigned to 13 treatments, comprising one spice-free control and 12 spice treatments resulting from four spices and three addition levels. Three patties were prepared in parallel for each treatment as processing subsamples. Each patty was prepared using 55.0 g of raw minced beef. Thus, 165.0 g of minced beef was prepared for each treatment within each independent replicate before being divided into three patties. The control group contained no added spice, whereas dried tangerine peel, rosemary, cinnamon, or clove powder was added individually at 0.1%, 0.5%, or 1.0% (*w*/*w*, based on the mass of raw minced beef). The intermediate level of 0.5% was selected with reference to Yu et al. [25], whereas 0.1% and 1.0% were included as lower and higher addition levels, respectively, to establish a concentration gradient and evaluate dose-dependent effects. All four spices were ground under identical conditions and passed through a 60-mesh sieve. For each treatment, the accurately weighed spice powder was divided into three portions and gradually sprinkled over the minced beef during manual mixing. After 1 min of premixing, the mixture was mechanically mixed in a household electric meat grinder at the highest setting for two 30-s cycles, with adhering material scraped and reincorporated between cycles, followed by 1 min of manual kneading. The same procedure was used for all treatments, and all equipment and utensils were thoroughly cleaned and dried between treatments to prevent cross-contamination. Beef patties were formed using a mold with a diameter of 90 mm and a thickness of 7 mm and then dry-marinated at 4 °C for 12 h. Before roasting, the patties were equilibrated to an initial center temperature of approximately 4 °C.

After dry marination, 1.10 g of sunflower oil, corresponding to 2.0% of the initial raw beef mass, was evenly brushed onto the surface of each patty. Each patty was placed on a baking tray lined with aluminum foil, with its lower surface in direct contact with the foil. The patties were roasted in a Midea PT3511 electric oven (Midea Group, Guangzhou, China) that had been preheated to 230 °C. The patties were roasted at 230 °C for 20 min and turned at 5, 10, and 15 min. During roasting, the oven temperature was monitored using a 4CH–20CH optical-fiber temperature transmitter (GND eTech Co. Ltd., Shanghai, China) to assess the temperature stability of the oven.

After roasting, the patties were cooled to room temperature. Cooking loss, color, and texture were determined using the three individual patties from each treatment before sample compositing. The results obtained from the three patties were averaged to provide one value for the corresponding treatment within each independent beef batch. After completion of these quality measurements, the remaining portions of the three patties from the same treatment and beef batch were combined, ground through a 5 mm plate, and thoroughly homogenized to obtain one composite sample for chemical analyses. Thus, each treatment generated one composite sample per independent beef batch. Each composite sample was divided into single-use aliquots sufficient for the individual chemical analyses, packed in food-grade PE composite vacuum bags, vacuum-sealed, and stored at −20 °C. For ALEs analysis, one aliquot of each homogenized composite sample was freeze-dried and ground into powder before analysis. All measurements were completed within 30 d. Before analysis, one aliquot was thawed at 4 °C for each assay, and the thawed aliquot was not refrozen. The three independently prepared beef batches represented the biological replicates (*n* = 3), whereas repeated measurements of the same aliquot were treated as technical replicates.

### 2.3. Determination of Basic Quality Attributes of Roasted Beef Patties

#### 2.3.1. Determination of Weight Loss During Roasting

Beef patties were weighed before roasting and recorded as *M*_0_, where the initial weight referred to the total weight of minced beef and added spices. After roasting, excess surface oil was removed using absorbent paper. The patties were then cooled to room temperature and weighed again as *M*_1_. The cooking loss of roasted beef patties was calculated using Equation (1):

(1) $$W=\frac{M0-M1}{M0}\times 100\%,$$

where *W* represents the cooking loss (%), *M*_0_ is the weight of the beef patty before roasting (g), and *M*1 is the weight of the beef patty after roasting and cooling (g).

#### 2.3.2. Color Measurement

Color measurements were performed on intact portions collected from each of the three patties before the remaining patty material was composited for chemical analyses. After roasting, beef patties were cooled to room temperature and cut into pieces suitable for the measurement window of the benchtop spectrophotometer (CM-5, Konica Minolta, Tokyo, Japan). Color parameters were measured using the CM-5 spectrophotometer and expressed in the CIE L* a* b* color space. L* represents lightness, a* represents redness–greenness, and b* represents yellowness–blueness. The L*, a* and b* values of each sample were recorded. The total color difference (*ΔE*) between each spice-treated group and its corresponding spice-free control group was calculated according to the CIE 1976 L*a*b* color space using Equation (2):

(2) $$\Delta E_{ab}^{*}=\sqrt{{\left(L_{t}^{*}-L_{c}^{*}\right)}^{2}+{\left(a_{t}^{*}-a_{c}^{*}\right)}^{2}+{\left(b_{t}^{*}-b_{c}^{*}\right)}^{2}},$$

where *L_t_*^∗^, *a_t_*^∗^, and *b_t_*^∗^ are the color coordinates of the spice-treated sample, and *L_c_*^∗^, *a_c_*^∗^, and *b_c_*∗ are those of the corresponding spice-free control sample. A larger *ΔE* value indicates a greater overall deviation in color from the spice-free control, whereas a smaller *ΔE* value indicates greater color similarity to the control. The measurements obtained from the three patties were averaged to provide one color value for each treatment within each independent beef batch.

#### 2.3.3. Texture Profile Analysis

The texture properties of the roasted beef patties were determined according to the method described by Li et al. [26]. After roasting, the beef patties were cooled to room temperature and cut into cubes measuring 10 mm × 10 mm × 10 mm. Compression tests were performed using a TA-XT plus texture analyzer (Stable Micro Systems, Godalming, UK) equipped with a P/50 cylindrical probe with a diameter of 50 mm.

The samples were subjected to two consecutive compression cycles. The test conditions were as follows: pre-test speed, 4.0 mm s^−1^; test speed, 3.0 mm s^−1^; post-test speed, 4.0 mm s^−1^; trigger force, 5.0 g; compression strain, 50%; and interval between the two compression cycles, 5.0 s. Texture parameters, including hardness, cohesiveness, gumminess, chewiness, and resilience, were recorded. Three texture measurements were performed for each treatment, and the mean value was used for subsequent analysis.

### 2.4. Determination of Dietary ALEs and Their Precursors

#### 2.4.1. Detection of Dietary ALEs

Although advanced glycation end products (AGEs) and ALEs originate from different carbonyl reaction pathways, both comprise structurally heterogeneous protein adducts, some of which exhibit characteristic fluorescence. Therefore, the Pronase-assisted hydrolysis procedure previously applied to protein-associated fluorescent AGEs and Maillard reaction products was adapted to release fluorescent products from the protein matrix Fluorescence and color as markers for the Maillard reaction in milk–cereal-based infant foods during storage [27]. The fluorescence detection wavelengths were selected according to the characteristic fluorescence of MDA-induced dietary ALEs established in our previous study [6].

Briefly, 0.100 g of freeze-dried roasted beef patty powder was mixed with 3.0 mL of Pronase E solution (0.375 mg mL^−1^; MedChemExpress, Monmouth Junction, NJ, USA) and incubated at 40 °C for 36 h for enzymatic hydrolysis. After hydrolysis, the mixture was cooled to room temperature and centrifuged at 5000 rpm for 10 min using a Sorvall ST16R refrigerated centrifuge (Thermo Fisher Scientific, Waltham, MA, USA). The resulting supernatant was collected and diluted 50-fold before fluorescence analysis.

Fluorescence intensity was measured using an F-7100 fluorescence spectrophotometer (Hitachi, Tokyo, Japan) at an excitation wavelength of 390 nm and an emission wavelength of 440 nm, with both the excitation and emission slit widths set at 5 nm. Each hydrolysate was measured three times, and the mean of the three technical readings was used as the analytical value for the corresponding biological replicate. The results were expressed as ALEs-associated relative fluorescence intensity. Because this fluorometric method does not distinguish individual ALEs structures, the measured fluorescence was used as an indicator of fluorescent dietary ALEs formation rather than as an absolute measure of total ALEs contents.

#### 2.4.2. Determination of MDA in Roasted Beef Patties

The MDA content of the roasted beef patties was determined using the thiobarbituric acid reactive substances method according to Kilic et al. [28] The results were expressed as milligrams of MDA per kilogram of roasted beef patty (mg MDA kg^−1^).

#### 2.4.3. Determination of MGO and GO in Roasted Beef Patties

GO and MGO in roasted beef patties were determined according to our previously published method, with minor modifications. Briefly, 5 g of homogenized roasted beef patty sample was accurately weighed into a 50 mL stoppered centrifuge tube and mixed with 10 mL of 0.1% (*v*/*v*) formic acid in ultrapure water. The mixture was vortexed at 2500 rpm for 3 min and centrifuged at 10,000× *g* for 20 min. The residue was extracted a second time under the same conditions, and the two supernatants were combined.

The combined supernatant was mixed with 10 mL of n-hexane, 0.5 mL of Carrez I solution containing 150 g L^−1^ potassium ferrocyanide trihydrate, and 0.5 mL of Carrez II solution containing 300 g L^−1^ zinc sulfate heptahydrate. After vortexing at 2500 rpm for 5 min, the mixture was centrifuged at 10,000× *g* for 10 min at 4 °C. The clear aqueous intermediate layer (3 mL) was transferred to an amber centrifuge tube, followed by the addition of 100 μL of o-phenylenediamine methanolic solution at 20 mg mL^−1^. Derivatization was performed in a shaking water bath at 60 °C and 100 rpm for 20 min.

Solid-phase extraction was performed using an Oasis HLB cartridge (Waters, Milford, MA, USA). The cartridge was conditioned and equilibrated sequentially with 5 mL of methanol and 5 mL of ultrapure water. The entire derivatized sample was loaded onto the cartridge, washed with 5 mL of ultrapure water, and then eluted with 3 mL of acetonitrile–water (1:1, *v*/*v*). The eluate was collected, filtered through a 0.22 μm organic microporous membrane, and analyzed by liquid chromatography–tandem mass spectrometry (LC–MS/MS).

GO and MGO were analyzed using a Waters Xevo triple-quadrupole mass spectrometer coupled to an ACQUITY UPLC I-Class system (Waters, Milford, MA, USA) operated in positive-ion mode. Chromatographic separation was performed using a Symmetry C18 column (150 mm × 4.6 mm, 5 μm; Waters) maintained at 30 °C. Mobile phase A was methanol, and mobile phase B was 0.1% formic acid in ultrapure water. The gradient program was as follows: 0 min, 30% A; 10 min, 60% A; and 12.0–12.1 min, 30% A. The flow rate was 0.5 mL min^−1^, and the injection volume was 10 μL. The mass spectrometric parameters for GO and MGO are shown in Table 1.

### 2.5. Determination of Total Phenolic and Flavonoid Contents and Antioxidant Capacity of Spice Extracts

Dried tangerine peel, rosemary, cinnamon and clove were separately ground and passed through a 60-mesh sieve. Spice powder (1 g) was accurately weighed and extracted with 30 mL of 60% ethanol by ultrasonic treatment at 70 °C for 1 h. The extract was centrifuged at 8000 r min^−1^ for 20 min, and the supernatant was collected and freeze-dried. The resulting extracts were used for subsequent determination of bioactive components.

The DPPH radical-scavenging activity and ABTS radical-scavenging activity were determined using the DPPH and ABTS assays, respectively. Sample solutions were prepared at concentrations of 0.05, 0.1, 0.5, 1, 2, 3 and 4 mg mL^−1^, with vitamin C used as the positive control. Absorbance was measured using a UV-1800 UV-visible spectrophotometer (Shimadzu, Kyoto, Japan) at 517 nm for the DPPH assay and 734 nm for the ABTS assay, and the radical-scavenging rates were calculated using Equations (3) and (4).

Ferric reducing antioxidant power (FRAP) was determined using the FRAP assay. The reaction mixture was incubated at 37 °C in the dark for 30 min, and absorbance was measured at 593 nm. FRAP values were calculated according to the FeSO_4_ standard curve. A higher FRAP value indicated a stronger reducing capacity of the spice extract.

Total phenolic content (TPC) was determined using the Folin–Ciocalteu method [29]. Gallic acid was used as the standard to establish the calibration curve, and absorbance was measured at 750 nm. TPC was calculated using Equation (5). Total flavonoid content (TFC) was determined using the NaNO_2_–Al(NO_3_)_3_–NaOH colorimetric method. Rutin was used as the standard to establish the calibration curve, and absorbance was measured at 510 nm. TFC was calculated using Equation (6). Each measurement was performed in triplicate.

(3) $$DPPH\;scavenging\;activity\left(\%\right)=\frac{1-\left(A_{x}-A_{x0}\right)}{A_{0}}\times 100\%,$$

where *A_x_* is the absorbance of the sample solution, *A_x_*_0_ is the background absorbance of the sample, and *A*_0_ is the absorbance of the control solution.

(4) $$ABTS\;scavenging\;activity(\%)=\frac{1-\left(B_{x}-B_{x0}\right)}{B_{0}}\times 100\%,$$

where *B_x_* is the absorbance of the sample solution, *B_x_*_0_ is the background absorbance of the sample, and *B*_0_ is the absorbance of the control solution.

(5) $$X_{1}(mg/g)=\frac{\rho_{1}\times V_{1}\times N_{1}}{m_{1}},$$

where *X*_1_ is the total phenolic content (mg g^−1^), $\rho_{1}$ is the mass concentration of total phenolics in the extract (mg mL^−1^), *V*_1_ is the extract volume, 30 mL, *m*_1_ is the mass of sample powder, 1 g; and *N*_1_ is the dilution factor.

(6) $$X_{2}(mg/g)=\frac{\rho_{2}\times V_{2}\times N_{2}}{m_{2}},$$

where *X*_2_ is the total flavonoid content (mg g^−1^); $\rho_{2}\;$ is the mass concentration of total flavonoids in the extract (mg mL^−1^); *V*_2_ is the extract volume, 30 mL; *m*_2_ is the mass of sample, 1 g; and *N*_2_ is the dilution factor.

### 2.6. Data Analysis and Statistical Analysis

Three independent replicate experiments were conducted using beef raw materials from three different batches. Each independent replicate started from patty preparation and roasting, and the preparation of all treatment groups and subsequent measurements were completed independently. Within each independent replicate, each index for the same sample was measured with at least three technical replicates. The mean value of the technical replicates was used as one observation for that independent replicate. Therefore, the independent sample size for each treatment group used in statistical analysis was three (*n* = 3). Results are expressed as the mean ± standard deviation of the three independent replicate experiments.

Statistical analysis was performed using SPSS 27.0 (IBM Corp., Armonk, NY, USA). Observations obtained from the independent replicate experiments were used as the statistical units. For each response variable, the spice-free control and all 12 spice-treated groups were included together in a single analysis. When the homogeneity-of-variance assumption was satisfied, one-way analysis of variance (ANOVA) followed by Tukey’s honestly significant difference (HSD) test was used for multiple comparisons. When the homogeneity-of-variance assumption was not satisfied, Welch’s ANOVA followed by the Games–Howell multiple-comparison test was applied. For ΔE, only the 12 spice-treated groups were analyzed because ΔE was calculated relative to the spice-free control. Statistical analyses were not performed separately according to spice concentration. Technical replicates were not treated as independent observations. A value of *p* < 0.05 was considered statistically significant. Figures were generated using Origin 2022 (OriginLab Corporation, Northampton, MA, USA). Chromatographic peak-area integration and part of the raw-data processing were performed using the software supplied with the instruments.

Pearson correlation analysis was used to evaluate the relationships among spice antioxidant properties, MDA, GO, MGO, ALEs-associated fluorescence and quality attributes. Principal component analysis (PCA) was performed using standardized treatment means to visualize the overall differences among treatments and the relationships among variables. Figures were generated using Origin 2022 (OriginLab Corporation, Northampton, MA, USA).

## 3. Results and Discussion

### 3.1. Effects of Different Spices on the Quality Attributes of Roasted Beef Patties

**Figure 1:**
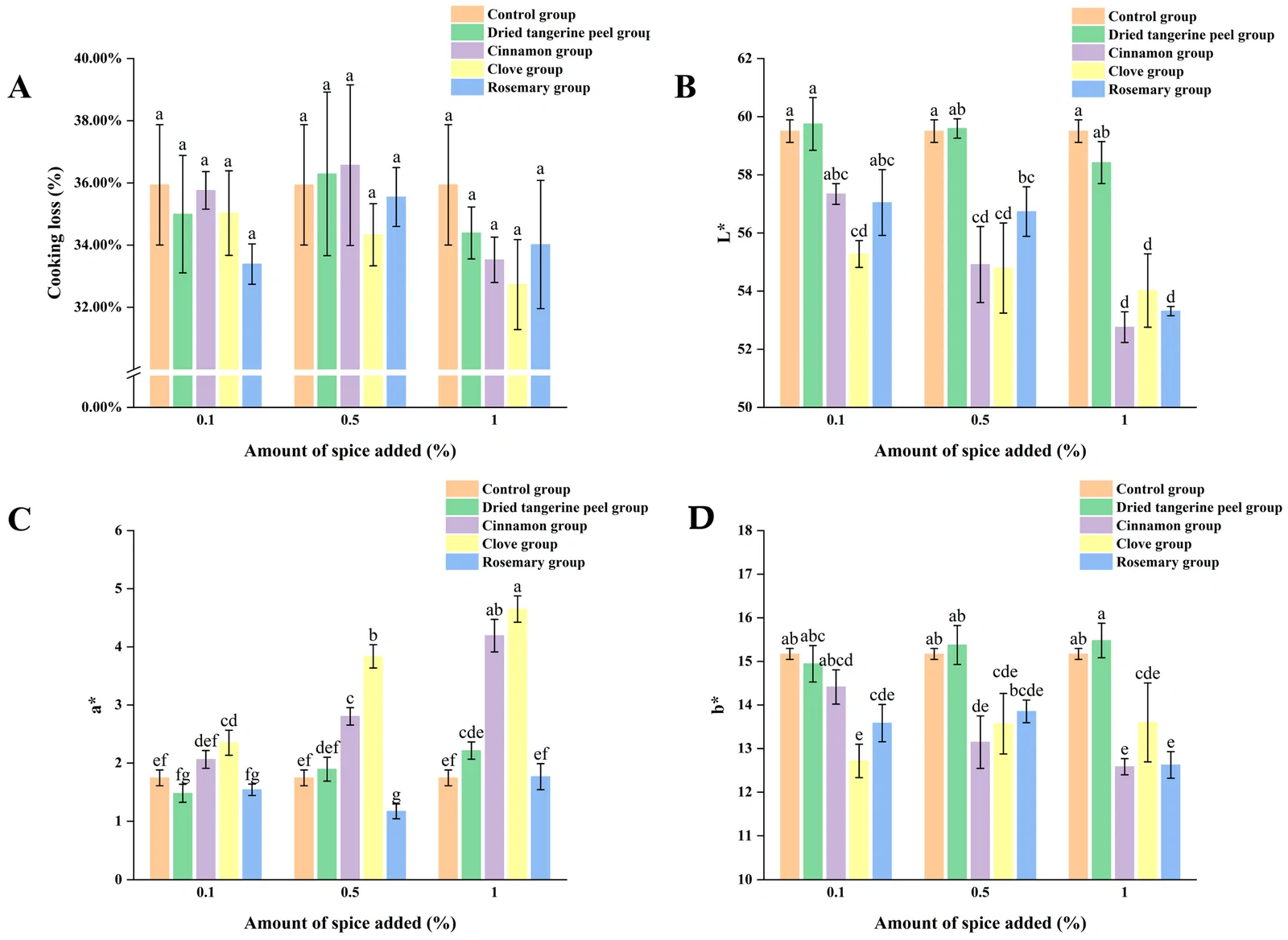
Effects of spice type and addition level on the cooking loss and color parameters of roasted beef patties. (**A**) Cooking loss; (**B**) L*; (**C**) a*; and (**D**) b*. Data are presented as mean ± standard deviation. For each response variable, the spice-free control and all 12 spice-treated groups were included together in a single one-way ANOVA followed by Tukey’s HSD multiple-comparison test. Different lowercase letters indicate significant differences among the 13 treatment groups (*p* < 0.05). Groups sharing at least one letter are not significantly different.

Spices are natural flavoring ingredients that can impart characteristic sensory attributes to roasted beef patties. At the same time, they may affect meat quality by regulating lipid oxidation, protein glycation and protein water-holding capacity [18]. Color and texture were measured to determine whether spice-based mitigation of ALE formation could be achieved without compromising product quality, while cooking loss was used to assess processing stability. Similar studies have also evaluated processing hazards and quality attributes together in spice-treated roasted beef patties [25]. Therefore, before evaluating their potential to mitigate ALEs formation, it is necessary to clarify how different spices influence the quality attributes of roasted beef patties. In this study, roasted beef patties treated with different spices were evaluated in terms of cooking loss, color and texture. The results are shown in Figure 1 and Table 2 and Table 3.

When all 13 treatment groups were included in a single statistical analysis, cooking loss did not differ significantly among treatments (*p* > 0.05; Figure 1A), indicating that neither spice type nor addition level markedly affected mass retention during roasting. In contrast, L, a, and b* were more responsive to spice addition, and the magnitude of these changes depended on both spice type and addition level (Figure 1B–D). The treatment effects were more evident for L* and a*, whereas the changes in b* were comparatively limited. As the spices were incorporated into the meat matrix before roasting, the observed instrumental color differences represent the net color response of the final products after spice incorporation and thermal processing, which may reflect the combined contributions of spice-derived pigments and spice-mediated changes in thermal browning and oxidation reactions.

Specifically, dried tangerine peel had the smallest effect on the overall color of the samples, with ΔE values of 0.76, 0.91, and 1.58 at addition levels of 0.1%, 0.5%, and 1.0%, respectively (Table 2). These relatively low ΔE values indicate that dried tangerine peel-treated patties remained closer in overall color to the spice-free control. Rosemary, clove and cinnamon changed the overall color of roasted beef patties to varying degrees, as reflected by their higher ΔE values. Moreover, the increasing ΔE values observed for rosemary and cinnamon with increasing addition levels indicate progressively greater overall color deviations from the control. Thus, the overall color response of roasted beef patties depended on both spice type and incorporation level.

Texture profile analysis showed that hardness, springiness, gumminess, and chewiness did not differ significantly among the 13 treatment groups (*p* > 0.05; Table 3). Although numerical variations were observed among treatments, these differences were not statistically significant. In contrast, cohesiveness differed significantly among treatments (*p* < 0.05). The 0.1% dried tangerine peel group showed higher cohesiveness than the cinnamon-, clove-, and rosemary-treated groups, whereas the control and the 0.5% and 1.0% dried tangerine peel groups showed intermediate values. These findings indicate that spice addition had a limited effect on most texture parameters under the present experimental conditions, with the main significant response being observed in cohesiveness.

Overall, different spices exerted distinct effects on the quality attributes of roasted beef patties. These effects were mainly observed in color and cohesiveness, whereas cooking loss and most other texture parameters were not significantly affected. The observed responses depended on both spice type and addition level, particularly for color and cohesiveness. These findings indicate that the four spices did not significantly impair cooking stability or most texture attributes, while modifying the appearance and cohesiveness of roasted beef patties to different extents. This provides a quality-related basis for further evaluating their regulatory effects on ALEs formation. Consistent with Yu et al. [25], these findings indicate that spice type and addition level should be selected by considering both hazard mitigation and product quality.

### 3.2. Effects of Different Spices on ALEs Formation in Roasted Beef Patties

In thermally processed high-fat and high-protein foods, protein–lipid interactions can promote the formation of ALEs, which are emerging as important food safety concerns in processed meat products [30]. Similar to heterocyclic amines and advanced glycation end-products generated through the Maillard reaction, dietary ALEs are increasingly recognized as potential hazards in thermally processed foods. Therefore, reducing ALEs accumulation in such foods is of practical importance.

To clarify the regulatory effects of different spices on ALEs formation in roasted beef patties, this study compared the effects of dried tangerine peel, cinnamon, clove and rosemary at different addition levels. Reactive carbonyl compounds derived from lipid oxidation can further covalently bind to proteins and amino acids, forming a series of relatively stable fluorescent complexes. Among them, some pyrrole- and pyridine-type cross-linked products show characteristic fluorescence signals at specific excitation wavelengths [6]. Previous studies have used the fluorescence response of MDA–protein reaction products to characterize the degree of modification and reaction kinetics, indicating that fluorescence signals can reflect the relative formation level of these products [31]. Therefore, bound fluorescence intensity was used in this study to characterize the relative accumulation of ALEs.

**Figure 2:**
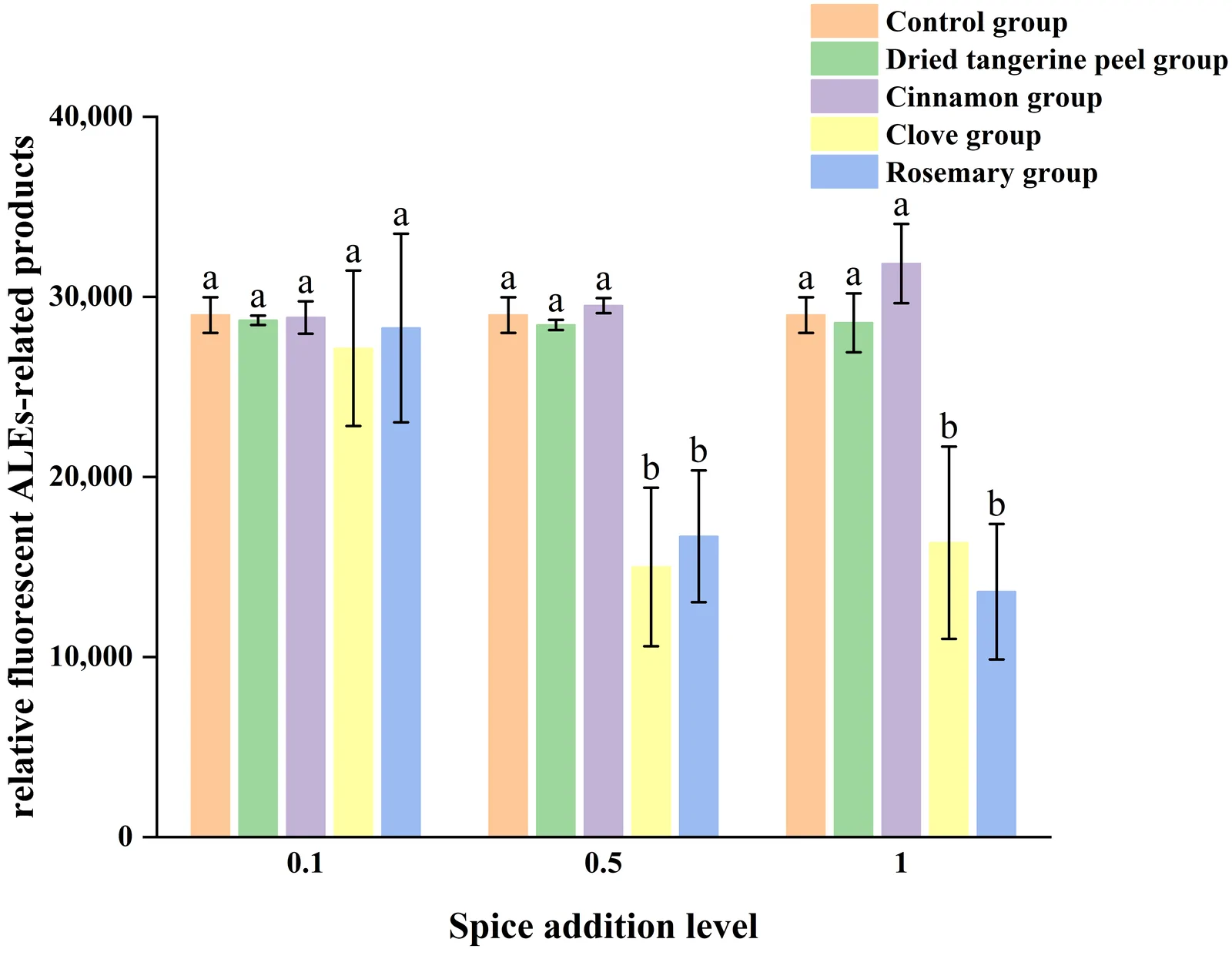
Effects of different spice addition levels on relative fluorescent ALEs-related products in roasted beef patties. Data are presented as mean ± standard deviation. The spice-free control and all 12 spice-treated groups were included together in a single one-way ANOVA followed by Tukey’s HSD multiple-comparison test. Different lowercase letters indicate significant differences among the 13 treatment groups (*p* < 0.05). Groups sharing at least one letter are not significantly different.

The results showed that different spices had distinct regulatory effects on ALEs accumulation in roasted beef patties, with a certain dose-dependent pattern. Clove and rosemary showed stronger and more stable inhibitory effects at medium and high addition levels, whereas dried tangerine peel and cinnamon showed relatively weaker effects. As shown in Figure 2, at the low addition level (0.1%), ALEs levels in the spice-treated groups were close to those in the control group, and the inhibitory effects were not yet evident. At medium and high addition levels (0.5% and 1.0%), the effects differed significantly among spices. At 0.5%, clove and rosemary significantly reduced ALEs-associated fluorescence compared with the control (*p* < 0.05), whereas dried tangerine peel and cinnamon did not differ significantly from the control (*p* > 0.05). These contrasting responses indicate that the four plant-derived spices differed in their ability to inhibit ALEs formation in roasted beef patties.

During thermal processing, changes in ALEs levels are closely related to precursor abundance, protein reactivity towards carbonyl compounds and the exposure of reactive sites [32]. Bioactive compounds in spices may inhibit ALEs formation by scavenging free radicals and trapping ALEs precursors through their antioxidant activity [25]. In addition, polyphenols can interact non-covalently with proteins, inducing changes in protein secondary and spatial structures [33], which may also affect ALEs formation. Therefore, the inhibitory effects of clove and rosemary on ALEs formation may result from multiple mechanisms, including reactive carbonyl trapping, free radical scavenging and modulation of protein structure.

Overall, clove and rosemary were the more effective inhibitory spices in this study. These findings suggest that both spices have potential value for mitigating processing-induced hazards and are feasible for practical application. For roasted meat products designed to balance flavor, appearance and health-related quality, clove and rosemary may serve as promising natural intervention ingredients.

### 3.3. Effects of Different Spices on ALEs Precursors in Roasted Beef Patties

ALEs are end-products formed through the modification of proteins by lipid oxidation products, and their accumulation is closely associated with the generation and reactivity of their precursors. Carbonyl compounds are considered key precursors that promote ALEs formation because of their relatively high stability and strong reactivity with proteins [34]. Among them, MDA is an important end-product of lipid peroxidation and a classical marker of lipid oxidation. GO and MGO are highly reactive carbonyl compounds that can further react with nucleophilic groups in proteins and promote ALEs formation. Therefore, this study determined the contents of MDA, GO and MGO in roasted beef patties treated with different spices to evaluate the effects of spices on representative carbonyl precursors of ALEs and to provide evidence for analyzing their potential inhibitory mechanisms.

**Figure 3:**
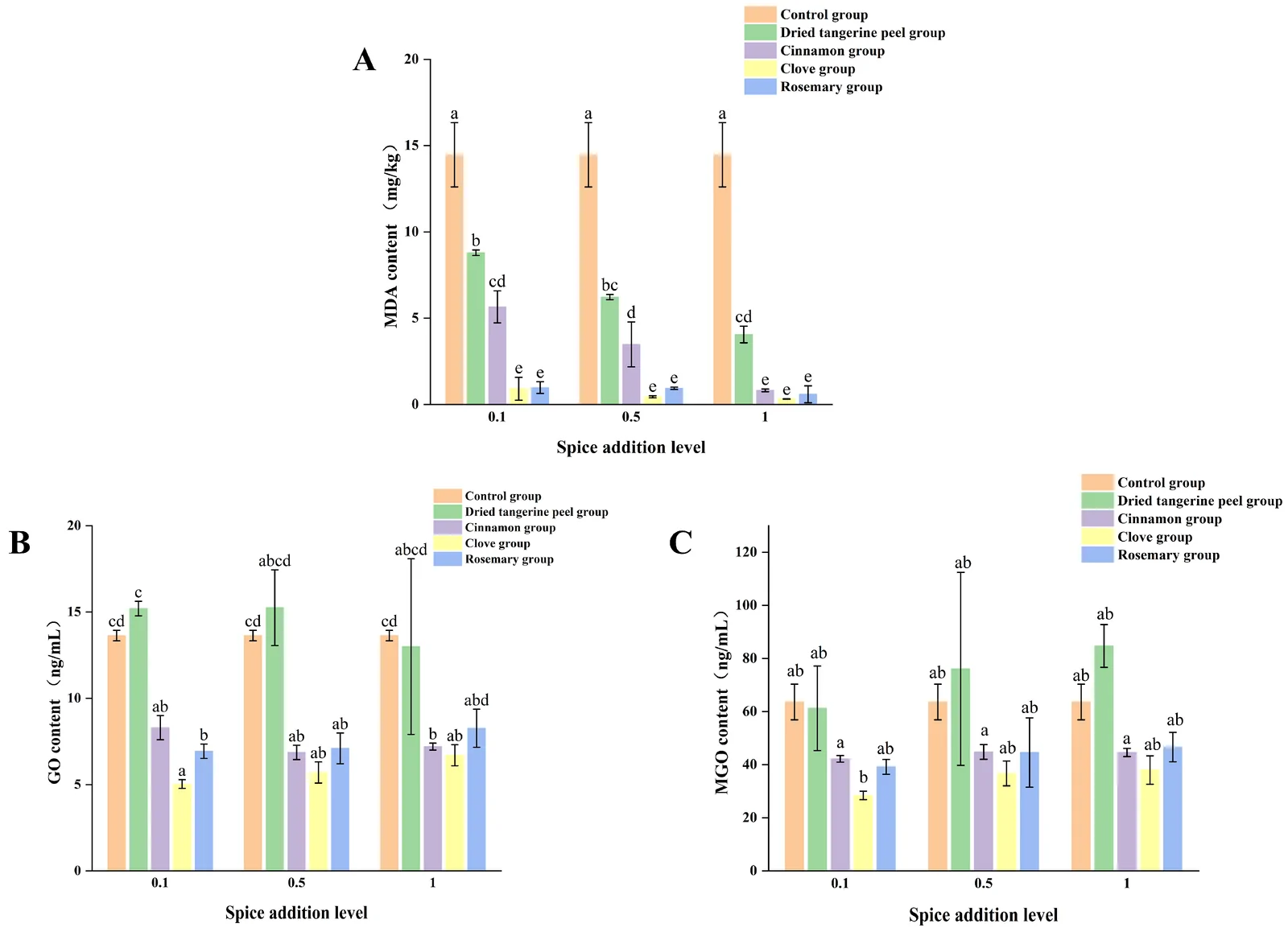
Effects of spice type and addition level on selected carbonyl compounds in roasted beef patties. (**A**) MDA content; (**B**) GO content; and (**C**) MGO content. Data are presented as mean ± standard deviation. All 13 treatment groups were compared together for each response variable. MDA was analyzed using one-way ANOVA followed by Tukey’s HSD multiple-comparison test. GO and MGO were analyzed using Welch’s ANOVA followed by the Games–Howell multiple-comparison test. Different lowercase letters indicate significant differences among the 13 treatment groups (*p* < 0.05). Groups sharing at least one letter are not significantly different.

The results showed that the effects of different spices on MDA, GO and MGO were not consistent (Figure 3). Overall, all four spices inhibited precursor accumulation to some extent, but their inhibitory strength and target profiles differed markedly. Clove and rosemary showed the strongest inhibitory effects on precursor accumulation, which was consistent with their stronger inhibition of ALEs formation. This suggests that suppression of precursor accumulation is likely an important basis for the ability of spices to limit ALEs formation.

However, the regulatory patterns differed among precursors. Changes in MDA were more consistent with the overall trend of ALEs inhibition (Figure 3A). The control patties showed high MDA levels after roasting. Campo et al. proposed approximately 2 mg MDA kg^−1^ as a sensory acceptability threshold for oxidized beef [35]. Our control exceeded this benchmark, indicating substantial lipid oxidation. However, TBARS thresholds depend on analytical method, meat matrix, processing conditions, and sensory protocol and are not universal safety limits [36]. Comparable values of 9.38–26.36 mg MDA kg^−1^ have been reported for oven-grilled beef, suggesting that intensive roasting at 230 °C for 20 min contributed to the high MDA level. In contrast, although GO and MGO are also reactive carbonyl compounds and can participate in protein modification, their changes were not fully aligned with ALEs variation (Figure 3B,C). This discrepancy may be explained by two factors. First, in complex thermally processed meat systems, GO and MGO formation is not driven solely by lipid oxidation, but may also be regulated by sugar degradation and other parallel reaction pathways. Their concentration changes may therefore not directly reflect the intensity of lipid oxidation. Second, as an important end-product of lipid peroxidation, MDA contains two reactive functional groups and is more prone than GO and MGO to undergo addition and cross-linking reactions with protein amino groups [37], thereby promoting ALEs formation. The comparatively strong response of MDA is consistent with previous studies showing that polyphenol-rich spices and plant ingredients can suppress lipid oxidation in meat systems. Li et al. reported that adding an antioxidant-rich spice mixture to hamburger meat reduced the MDA concentration of the cooked product by 71% [38]. Sobral et al. also found that oregano inhibited lipid and protein oxidation in chicken burgers during cooking and simulated gastrointestinal digestion [39]. Although conducted under simulated digestion rather than roasting alone, Li et al. showed that fiber-bound polyphenols from highland barley reduced MDA and other aldehydic lipid-oxidation products during the digestion of cooked pork, while Aksoy et al. reported lower MDA formation in meat products treated with polyphenol-rich hardaliye [40,41]. These studies support the capacity of plant phenolics to limit the formation or accumulation of reactive lipid-derived carbonyl compounds.

Nevertheless, antioxidant effects are dependent on the food matrix and the oxidation endpoint examined. Leung et al. found that rosemary-infused oil reduced hydroperoxide and 4-HNE formation in pan-fried salmon but did not suppress all measured oxidized polyunsaturated fatty-acid products [42]. In addition, Ali et al. demonstrated that production system and muscle type affected the susceptibility of chicken meat to lipid and protein oxidation during heating and digestion [43]. These observations help explain why MDA, GO and MGO did not respond uniformly in the present study. They also indicate that the effectiveness of a spice cannot be predicted solely from its in vitro antioxidant capacity, but is influenced by the meat matrix, heating conditions, spice form and concentration, and the chemical marker selected.

### 3.4. Total Phenolic and Flavonoid Contents and Antioxidant Capacity of Different Spices

To further explore the relationship between the inhibitory effects of different spices on ALEs and their precursors and their phenolic and flavonoid contents and antioxidant potential, this study determined the total flavonoid content, total phenolic content and in vitro antioxidant activity of dried tangerine peel, cinnamon, clove and rosemary. As shown in Table 4, the four spices differed significantly in total phenolic and flavonoid contents and antioxidant capacity. Clove exhibited the highest total flavonoid and total phenolic contents, at 9.49 ± 0.05 mg g^−1^ and a total phenolic content of 143.86 ± 3.72 mg g^−1^. Both values were significantly higher than those of the other three spices, which may be closely associated with the strong inhibitory effect of clove on ALEs formation. Rosemary and cinnamon showed intermediate levels, but their values were markedly lower than those of clove. Dried tangerine peel had the lowest total flavonoid and total phenolic contents, significantly lower than those of the other three spices.

In terms of antioxidant activity, clove and cinnamon showed higher radical-scavenging activity, indicating stronger hydrogen-donating capacity and free radical-quenching ability. The ABTS and DPPH radical-scavenging rates of rosemary were generally lower than those of clove and cinnamon, but remained markedly higher than those of dried tangerine peel. The FRAP results further showed that clove had the strongest electron-donating capacity and reducing potential, with a FRAP value of 1.39 ± 0.06 μmol mL^−1^, significantly higher than those of cinnamon, rosemary and dried tangerine peel.

Overall, the four spices exhibited distinct differences in total phenolic and flavonoid contents and antioxidant capacity. Clove showed the highest total phenolic and flavonoid contents and the strongest antioxidant capacity, whereas dried tangerine peel showed relatively lower values. Although the antioxidant responses of cinnamon and rosemary varied among different assays, their antioxidant properties were generally higher than those of dried tangerine peel. These differences may be associated with the characteristic bioactive constituents reported for each spice. Previous studies have shown that dried tangerine peel contains flavonoids such as hesperidin, nobiletin and tangeretin [44], while procyanidins and cinnamaldehyde are considered important contributors to the antioxidant properties of cinnamon [45]. Eugenol and gallic acid have been reported as major antioxidant constituents of clove [46], whereas carnosic acid, carnosol and rosmarinic acid are characteristic antioxidant compounds in rosemary [47]. These compositional differences may explain why cinnamon was less effective than clove and rosemary in inhibiting ALE formation. Although cinnamon and clove showed similar ABTS and DPPH activities, clove had markedly higher total phenolic and flavonoid contents and a higher FRAP value. Moreover, cinnamon’s higher in vitro antioxidant indices than rosemary did not translate into stronger inhibition in the beef patty matrix, possibly because rosemary’s characteristic antioxidants more effectively suppressed lipid oxidation and reactive carbonyl formation. Consistent with the closer association between MDA and ALE-associated fluorescence, cinnamon’s weaker effect likely reflected differences in antioxidant content and pathway-specific activity rather than insufficient in vitro antioxidant capacity. Given its highest total phenolic and flavonoid contents together with strong antioxidant capacity, clove may possess greater potential to suppress ALEs formation during thermal processing by scavenging free radicals, inhibiting lipid oxidation and limiting the generation of reactive carbonyl compounds.

### 3.5. Association Structure Among Spice Antioxidant Properties, Precursors, ALEs and Quality Attributes

To clarify the relationships among spice antioxidant activity, ALEs levels, precursor accumulation and roasted beef patty quality, Pearson correlation analysis and PCA were used for integrated evaluation of the measured indicators.

**Figure 4:**
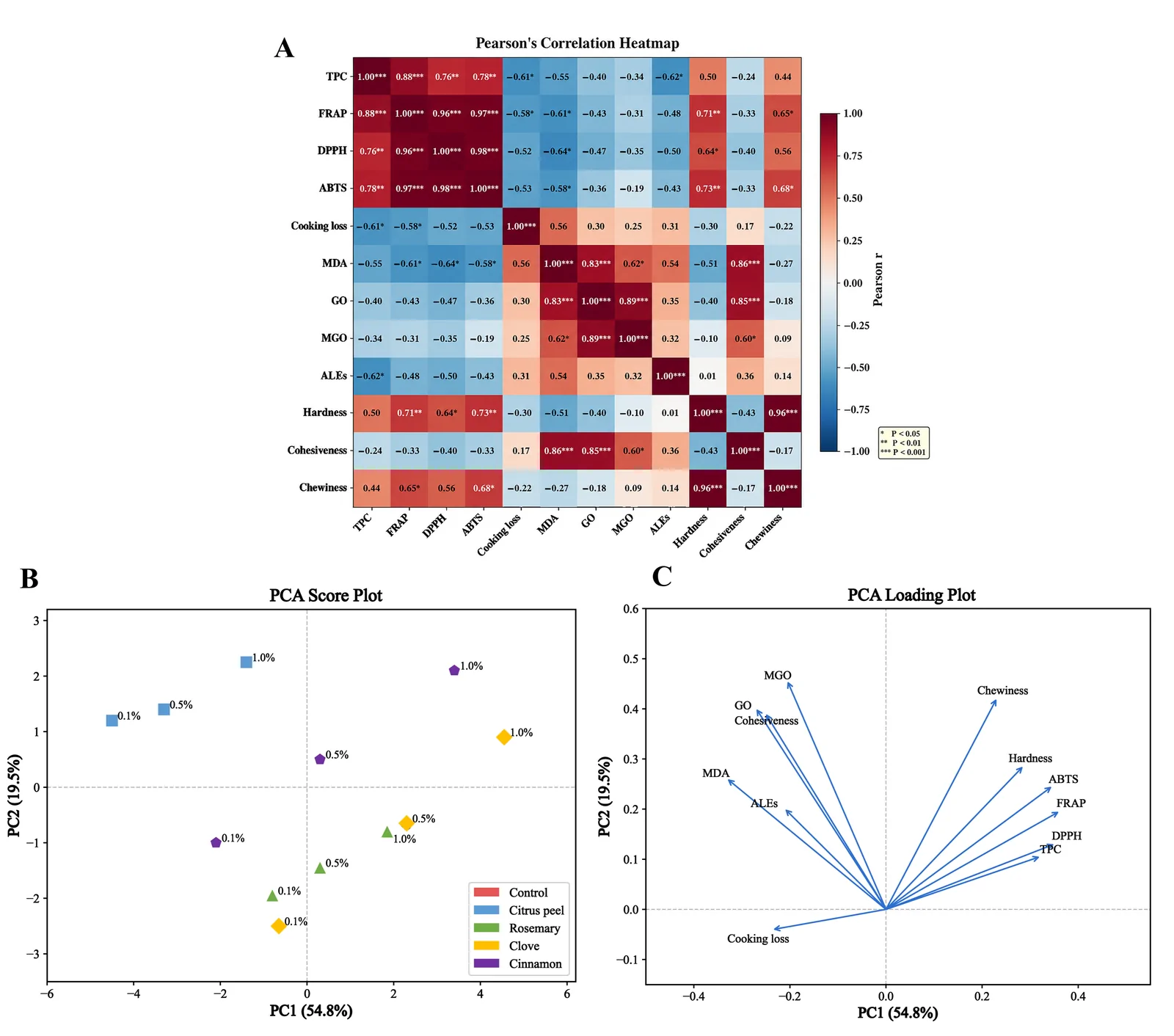
Correlation analysis and principal component analysis of antioxidant properties, selected carbonyl compounds and fluorescent ALE-related products. (**A**) Pearson correlation analysis; (**B**) PCA score plot; (**C**) PCA loading plot.

Pearson correlation analysis showed that antioxidant indicators generally changed in the opposite direction to precursor and ALEs levels. TPC, FRAP, DPPH and ABTS all showed negative correlations with ALEs, indicating that spices with richer phenolic bioactive compounds and stronger antioxidant capacity were associated with lower ALEs accumulation in the samples. Meanwhile, FRAP, DPPH and ABTS were clearly negatively correlated with MDA, suggesting that stronger antioxidant capacity reduced the extent of lipid peroxidation (Figure 4A). Therefore, clove and rosemary, indicating an association between antioxidant capacity and lower MDA accumulation (Figure 4A). Nevertheless, the effects of individual spices were not fully explained by their in vitro antioxidant capacity. Cinnamon exhibited antioxidant capacity comparable to or higher than rosemary, but showed weaker inhibition of ALE-associated fluorescence. Given that MDA varied more consistently with ALEs than GO and MGO, the spice-dependent inhibition of ALE formation was likely related to both antioxidant properties and the regulation of carbonyl precursor accumulation. Regarding quality attributes, cooking loss was not significantly correlated with the antioxidant indicators, consistent with the absence of significant differences in cooking loss among the treatments (Figure 1A).

The PCA results further revealed the overall relationships among the measured indicators. As shown in the score plot (Figure 4B), samples treated with different spices and addition levels were clearly separated in the PCA space. Dried tangerine peel-treated samples were mainly distributed in the negative region of PC1, indicating that their effect on reducing oxidation-derived hazard accumulation was relatively limited. In contrast, rosemary- and clove-treated samples gradually shifted towards the positive region of PC1 as the addition level increased. In particular, samples with high addition levels were closer to the region where antioxidant indicators were located, indicating that these treatments were positioned in the direction associated with lower accumulation of oxidation-derived hazards.

The loading plot (Figure 4C) further showed that TPC, ABTS, DPPH and FRAP clustered in the positive direction of PC1, whereas MDA, GO, MGO and ALEs were mainly distributed in the negative direction of PC1. This indicates an overall opposite trend between antioxidant indicators and oxidation-derived hazard indicators. Meanwhile, hardness and chewiness were positioned relatively close to the antioxidant indicators, whereas cohesiveness was closer to GO and MGO. These results suggest that the regulatory effects of spices were reflected not only in changes in precursors and ALEs, but also in the modulation of texture properties.

Together, Pearson correlation analysis and PCA demonstrate that the phenolic and flavonoid contents and antioxidant potential of spices provide an important chemical basis for their differential effects. These differences further influence lipid oxidation precursor accumulation and the overall oxidative environment, ultimately leading to distinct levels of ALEs formation.

## 4. Conclusions

This study demonstrated that the effects of spices on fluorescent ALE-associated product formation in roasted beef patties were spice- and dose-dependent. Clove and rosemary exerted stronger inhibitory effects, particularly at addition levels of 0.5% and 1.0%. Their effects were associated with reduced accumulation of reactive carbonyl precursors, especially MDA, whereas the responses of GO and MGO varied among spice treatments. Clove exhibited the highest total phenolic and flavonoid contents and strong overall antioxidant potential. However, cinnamon showed antioxidant capacity comparable to or higher than rosemary in several in vitro assays but did not exhibit correspondingly strong inhibition of ALE-associated fluorescence, indicating that in vitro antioxidant capacity alone cannot fully explain the spice-dependent responses. Changes in MDA were more consistent with those in ALE-associated fluorescence than the changes in GO or MGO, suggesting that the inhibition of ALE-associated product formation may be associated with both antioxidant properties and the regulation of carbonyl-precursor accumulation within the meat matrix. Spice addition did not significantly affect cooking loss, although color and texture were modified to different extents. Overall, clove and rosemary show potential as natural ingredients for mitigating lipid oxidation-derived protein modification in roasted meat. Further studies using targeted high-resolution mass spectrometry are required to identify specific ALE structures and verify the underlying molecular mechanisms.

## Figures and Tables

**Table 1:** Mass spectrometric parameters for GO and MGO.

	Time (min)	Flow Rate (mL min^−1^)	%A	%B	Injection Volume/μL	Curve
1	Initial	0.500	30.0	70.0	10	Initial
2	10.00	0.500	60.0	40.0	10	6
3	12.00	0.500	30.0	70.0	10	6
4	15.00	0.500	30.0	70.0	10	6

**Table 2:** Effect of different spices on the color difference of roasted beef patties.

Group	0.1% Treatment Group	0.5% Treatment Group	1.0% Treatment Group
Rosemary group	2.44 ^a^	2.79 ^ab^	6.21 ^c^
Clove group	4.3 ^b^	5.02 ^c^	5.52 ^cd^
Cinnamon group	2.2 ^a^	4.73 ^bc^	6.76 ^d^
Dried tangerine peel group	0.76 ^e^	0.91 ^e^	1.58 ^f^

Different lowercase letters indicate significant differences among the difference groups (*p* < 0.05).

**Table 3:** Effect of different spices on the texture of roasted beef patties.

Group	Hardness (N)	Springiness	Gumminess (N)	Cohesiveness	Chewiness (g)
Control group	55.35 ± 11.54 ^a^	0.87 ± 0.01 ^a^	42.62 ± 7.97 ^a^	0.77 ± 0.02 ^ab^	36.88 ± 6.96 ^a^
0.1% rosemary group	38.24 ± 9.03 ^a^	0.83 ± 0.04 ^a^	27.05 ± 6.96 ^a^	0.71 ± 0.02 ^b^	22.38 ± 4.98 ^a^
0.1% clove group	38.24 ± 9.03 ^a^	0.83 ± 0.04 ^a^	27.80 ± 17.43 ^a^	0.71 ± 0.02 ^b^	22.38 ± 4.98 ^a^
0.1% cinnamon group	37.37 ± 22.04 ^a^	0.88 ± 0.03 ^a^	28.59 ± 13.77 ^a^	0.73 ± 0.03 ^b^	24.21 ± 15.10 ^a^
0.1% dried tangerine peel group	25.73 ± 10.91 ^a^	0.94 ± 0.02 ^a^	22.50 ± 11.37 ^a^	0.86 ± 0.08 ^a^	21.36 ± 11.17 ^a^
0.5% rosemary group	40.80 ± 20.07 ^a^	0.88 ± 0.01 ^a^	28.59 ± 13.77 ^a^	0.70 ± 0.02 ^b^	24.98 ± 11.74 ^a^
0.5% clove group	54.42 ± 13.47 ^a^	0.90 ± 0.07 ^a^	38.59 ± 10.90 ^a^	0.70 ± 0.03 ^b^	34.09 ± 7.45 ^a^
0.5% cinnamon group	55.57 ± 20.54 ^a^	0.91 ± 0.08 ^a^	40.06 ± 14.95 ^a^	0.72 ± 0.02 ^b^	36.16 ± 12.18 ^a^
0.5% dried tangerine peel group	39.05 ± 6.82 ^a^	0.88 ± 0.04 ^a^	30.10 ± 6.33 ^a^	0.77 ± 0.03 ^ab^	26.61 ± 6.77 ^a^
1.0% rosemary group	38.55 ± 6.93 ^a^	0.84 ± 0.01 ^a^	27.16 ± 4.55 ^a^	0.71 ± 0.03 ^b^	22.88 ± 3.77 ^a^
1.0% clove group	53.43 ± 12.35 ^a^	0.87 ± 0.04 ^a^	39.33 ± 8.90 ^a^	0.74 ± 0.01 ^b^	33.97 ± 6.43 ^a^
1.0% cinnamon group	67.52 ± 13.65 ^a^	0.91 ± 0.08 ^a^	48.78 ± 10.55 ^a^	0.72 ± 0.02 ^b^	44.04 ± 7.84 ^a^
1.0% dried tangerine peel group	52.12 ± 5.07 ^a^	0.89 ± 0.01 ^a^	39.58 ± 3.30 ^a^	0.76 ± 0.01 ^ab^	35.39 ± 2.64 ^a^

Different lowercase letters indicate significant differences among the difference groups (*p* < 0.05).

**Table 4:** Total flavonoid content, total phenolic content and antioxidant activity of different spices.

Group	Flavonoid Content (mg g^−1^)	Total Phenolic Content (mg g^−1^)	ABTS Scavenging Activity (%)	DPPH Scavenging Activity (%)	FRAP Value (μmol mL^−1^)
Dried tangerine peel group	0.56 ± 0.02 ^c^	16.21 ± 0.97 ^c^	35.13 ± 4.54 ^c^	11.02 ± 3.18 ^c^	0.26 ± 0.01 ^d^
Cinnamon group	5.76 ± 0.09 ^b^	43.83 ± 1.82 ^b^	95.16 ± 1.91 ^a^	72.28 ± 1.53 ^a^	1.09 ± 0.03 ^b^
Clove group	9.49 ± 0.05 ^a^	143.86 ± 3.72 ^a^	95.48 ± 0.46 ^a^	72.97 ± 1.75 ^a^	1.39 ± 0.06 ^a^
Rosemary group	5.83 ± 0.17 ^b^	45.55 ± 2.46 ^b^	76.15 ± 4.35 ^b^	65.68 ± 2.48 ^b^	0.80 ± 0.03 ^c^

Different lowercase letters indicate significant differences among the difference groups (*p* < 0.05).

## Data Availability

The data presented in this study are available on request from the corresponding authors due to intellectual property protection (patent-related) considerations, the raw data are not publicly available at this stage.

## Abbreviations

The following abbreviations are used in this manuscript:

ALEs	Advanced lipoxidation end products
AGEs	Advanced glycation end products
MDA	Malondialdehyde
GO	Glyoxal
MGO	Methylglyoxal
UPLC-MS/MS	Ultra-performance liquid chromatography–tandem mass spectrometry
HPLC	High-performance liquid chromatography
LC-MS/MS	Liquid chromatography–tandem mass spectrometry
SPE	Solid-phase extraction
TCA	Trichloroacetic acid
DPPH	2,2-diphenyl-1-picrylhydrazyl
ABTS	2,2′-azinobis-(3-ethylbenzothiazoline-6-sulfonic acid)
FRAP	Ferric reducing antioxidant power
TPC	Total phenolic content
TFC	Total flavonoid content
PCA	Principal component analysis
ANOVA	Analysis of variance
HSD	Honestly significant difference
